# Molecular Biophysics of Class A G Protein Coupled Receptors–Lipids Interactome at a Glance—Highlights from the A_2A_ Adenosine Receptor

**DOI:** 10.3390/biom13060957

**Published:** 2023-06-07

**Authors:** Efpraxia Tzortzini, Antonios Kolocouris

**Affiliations:** Laboratory of Medicinal Chemistry, Section of Pharmaceutical Chemistry, Department of Pharmacy, School of Health Sciences, National and Kapodistrian University of Athens, 15771 Athens, Greece; evtzortz@pharm.uoa.gr

**Keywords:** A_2A_ adenosine receptor, cholesterol, coarse graining, GPCR, molecular dynamics, PIP_2_

## Abstract

G protein-coupled receptors (GPCRs) are embedded in phospholipid membrane bilayers with cholesterol representing 34% of the total lipid content in mammalian plasma membranes. Membrane lipids interact with GPCRs structures and modulate their function and drug-stimulated signaling through conformational selection. It has been shown that anionic phospholipids form strong interactions between positively charged residues in the G protein and the TM5-TM6-TM 7 cytoplasmic interface of class A GPCRs stabilizing the signaling GPCR-G complex. Cholesterol with a high content in plasma membranes can be identified in more specific sites in the transmembrane region of GPCRs, such as the Cholesterol Consensus Motif (CCM) and Cholesterol Recognition Amino Acid Consensus (CRAC) motifs and other receptor dependent and receptor state dependent sites. Experimental biophysical methods, atomistic (AA) MD simulations and coarse-grained (CG) molecular dynamics simulations have been applied to investigate these interactions. We emphasized here the impact of phosphatidyl inositol-4,5-bisphosphate (PtdIns(4,5)P_2_ or PIP_2_), a minor phospholipid component and of cholesterol on the function-related conformational equilibria of the human A_2A_ adenosine receptor (A_2A_R), a representative receptor in class A GPCR. Several GPCRs of class A interacted with PIP_2_ and cholesterol and in many cases the mechanism of the modulation of their function remains unknown. This review provides a helpful comprehensive overview for biophysics that enter the field of GPCRs-lipid systems.

## 1. Lipids in Biological Membranes

Membranes are crucial for life as they form the barriers that separate cells and enveloped viruses from their environment and arrange them in sections forming different organelles. Their composition depends on the cell type and age, metabolic state, and spatial location. They are lipid bilayers composed of a complex mixture of various amphipathic lipid species distributed asymmetrically between the two leaflets. More than a thousand types of lipids have been identified in living cells and the requirements for barrier functions are not enough to explain the enormous degree of structural diversity of lipids, which ranges from subtle differences such as the position of a double bond in the acyl chain, to major ones such as different backbones, i.e., different alkyl chain lengths which is the main lipophilic part of the lipid [1,2,3].

Lipids have been found to serve major functions in cells including membrane structural components [2], energy and heat sources [3], protein recruitment platforms [4], signaling molecules [5] and substrates for translational protein-lipid modifications [6]. Nowadays, it is evident that the lipid component of membranes is an essential player in understanding the mechanism of action and targeting of many drugs [7,8], and the importance of lipid-protein interactions is recognized [9].

We can obtain a rough idea about membrane organization and structure through the fluid mosaic model, which was proposed in 1972 and describes the structure of the membrane as a bilayer of freely laterally diffusing polar lipid forming a highly hydrophobic core and acting as solvent for membrane proteins [10]. Major membrane lipids are classified into glycerophospholipids, sphingolipids and sterols. Key feature of biological membranes is their asymmetry, with the individual monolayers that make up the bilayer having distinct lipid compositions and associated functional implications [11].

Plasma membranes concentrate 80–90% of the total cell cholesterol content. The cytoplasmic leaflet of the plasma membrane of mammalian cells usually contains more phosphatidyletholamine (PE) and phospahotidylserine (PS) when compared with the outer leaflet which is rich in shpingolipids [12]. This asymmetric nature of the cell membranes was known for some time before the fluid mosaic membrane model was published [13,14] and it is proposed that this asymmetric nature is one of the five major principles that govern the membrane structure [14]. Furthermore, as the existence of several phospholipid transporters for maintaining the proper phospholipid asymmetries in the cell membrane implies, this asymmetry is functionally essential for the cells and its disruption is generally associated with cell activation and with pathogenic conditions [15,16,17]. One major component of membranes in mammalian cells is cholesterol, an isoprenoid-derived rigid lipid molecule that is essential in sustaining the structural stability of the membrane as well as its fluidity and can also modulate biological processes [18]. The plasma membrane also includes as a minor component the anionic phospholipid, phosphatidyl inositol-4,5-bisphosphate (PtdIns(4,5)P_2_ or PIP_2._).

Except for lipids that compose membranes, proteins are also embedded into them, resulting in a complex protein-lipid interactome, and of particular interest is that the bilayer lipid mixture and certain lipid molecules can modulate the function of integral proteins by binding to specific sites on them and altering their structure and function [19,20,21,22,23,24,25,26]. The most common type of integral membrane protein is the transmembrane (TM) protein, which spans the entire biological membrane. Single-pass membrane proteins cross the membrane only once, while multi-pass membrane proteins weave in and out, crossing several times.

Summarizing, lipids influence both the structure and function of integral membrane proteins, and membrane proteins should be considered as lipoprotein complexes.

The theme of this article is the molecular biophysics of G protein coupled receptors (GPCRs)-lipid interactome. While there are excellent reviews from experts [23,26,27,28,29,30] this review was directed at newcomer scientists willing to be informed for this field. We emphasized the effect of PIP_2_ and cholesterol on human A_2A_ adenosine receptor (A_2A_R), function a representative class A GPCR. As we discussed, such critical interactions with cholesterol and PIP_2_ have been identified for other class A GPCRs and the structural basis of these interactions gives opportunities for the design of allosteric drugs targeting these receptors. We mentioned the group leaders that solved GPCRs experimental structures or involved with class A GPCRs—lipid interactions. However, in many cases although these lipids affected the function of many membrane proteins including GPCRs, according to biophysical/biochemical assays, the mechanism of the function modulation remains unknown. This is a challenging research field for scientists involved with experimental methods or molecular dynamics (MD) simulations. Results from carefully performed MD simulations are useful for both predictions but also for the interpretation of experimental findings.

## 2. Membrane Proteins—Lipids Interactome

### 2.1. Interactions between Membrane Lipids and Membrane Proteins

Lipid environment and plasma membrane composition [19,20,21,22,23,24,25,26,31,32] are known to modulate the function of a range of membrane proteins [27], e.g., GPCRs, [23,26] potassium channels [20,21], receptor tyrosine kinases (RTKs) [22] and certain P-type ATPases [33,34]. Lipids can influence several aspects of a membrane protein behavior by modulating protein-protein interactions [35,36,37], altering cellular localization by recruiting a protein to spatially defined regions of a membrane and ultimately affecting the functional activity of the protein.

Lipids interact with membrane proteins via multiple modes. The presence of integral membrane proteins may induce formation of a lipid ‘annulus’ around the protein. Due to interactions with the protein, lipids within this annulus exhibit decreased motional freedom compared to their non-interacting bulk counterparts and can be detected by EPR spectroscopy [38,39]. This immobilizing effect of the protein to the surrounding lipids may extend beyond the first shell of directly interacting annular lipids, leading to further outer shells with a lesser extent of lipid immobilization, as suggested by MD simulations [40,41].

In addition, certain lipid species may bind to specific sites on the membrane protein surface—often described as ‘non-annular’ lipids. Binding is driven by formation of physicochemical interactions between the lipid and protein surface, as well as by complementary geometry deep clefts (or cavities) on the protein surface [42] or binding at the interface between protein momomers [43]. The direct interactions by lipids in non-annular sites are characterized by lack of accessibility to the annular lipids, *i.e.*, these sites cannot be displaced by competition with annular lipids. Binding sites may tightly coordinate the lipid [44], or act to cause weaker and more dynamic localization [45]. Lipids that are co-crystallized with membrane proteins (and therefore remain preserved even in the crystal structure) belong to the class of non-annular (or sometimes termed as “co-factor”) lipids. Efforts have been made to describe general features of lipid binding sites and sequence interaction motifs, as in the case of cholesterol binding motifs [46].

There is evidence that lipids can be allosteric modulators, i.e., the lipid binds to a site on the receptor that is separate to where the signal molecule binds, of membrane protein structure and activation. Thus, lipids may modulate the functional activity of a membrane receptor either by making direct interactions, affecting the signal molecule binding pocket directly [47] or causing long-range allosteric events [48], or by affecting the general physical properties of the membrane.

More recently, studies have suggested lipid molecules may exit the bulk lipid phase of the membrane, and laterally enter the core of integral membranes proteins. This can include the entry of entire lipid molecules into the orthosteric binding pocket of G protein-coupled receptors (GPCRs) [49,50] as well as the protrusion of tails into the selectivity filter of ion channels [51].

### 2.2. Identification of Interaction Sites of Lipids in Integral Membrane Proteins with Experimental Methods

Experimental study of integral membrane proteins is challenging because of their location, embedded in the biological membrane. Detergent solubilization is widely used to extract integral proteins from the membrane although this procedure usually strips integral membrane proteins from their molecular partners: lipids. Advances in lipidomics [52] and in structural biology of membrane proteins [53,54,55] over the past decade have revealed some of the complexities of the cell membranes composition and have showed the lipid modulation of integral proteins.

Many high-resolution structures of membrane proteins with bound lipids have resolved by X-ray crystallography [56] or cryo-electron microscopy (cryo-EM [57] (reviewed in reference [58]) and this structural identification of specific binding sites of lipids aids in understanding of lipid modulation of protein function [59]. Examples of other biophysical experimental techniques that allow to probe lipid interactions with membrane proteins are NMR spectroscopy [60], fluorescent spectroscopy [61], and mass spectroscopy [35,62,63].

### 2.3. Identification of Interaction Sites of Lipids in Integral Membrane Proteins with MD Simulations

Multiscale simulations for studying membrane protein biophysics have become a standard method as computer power has increased by at least 4 orders of magnitude over the past 20 years [64,65,66].

MD simulations allow for the identification of binding sites, some of them presumably weaker, that are not often observed by X-ray crystallography [56] or cryo-EM. The atomistic (AA) MD simulation-based characterization is often made in terms of occupancy of a specific lipid in a specific protein site observed in the trajectory and it does not allow qualitative assessment of binding affinity, which can only be obtained using more advanced free energy calculations [67]. However, the computational cost of the AA MD simulations is such that length scales beyond microseconds are not currently readily accessible [68] and as the complexity of simulated systems increases the simulation time required to obtain converged averages also increases. This has prompted the development of the more approximate coarse-grained (CG) force fields of membrane lipids and proteins in MD simulations [69,70] in which groups of atoms are represented as single particles and that way reducing both the number of particles in the system and the chemical specificity and level of detail. Consequently, the computational demand for running CG MD simulations is reduced and thus allows access to longer time and length scales simulations of proteins in membranes, with the caveat of the reduced accuracy in the description of the underlying biological interactions being probed. CG MD simulations can thus allow significantly for much longer simulations. This enables to sample more efficiently the diffusion and binding of lipids, to membrane proteins [71,72,73,74] enhancing lipid exploration of the protein surface and candidate binding sites, whilst sacrificing the finer detail of lipid-protein interactions.

Advantages due to the increase in the processing power and algorithms efficiency as well as the ability to reversibly convert between CG and AA modes compete disadvantages related with sampling of the conformational space of atomic models arising from the timescale limitations [75,76]. Thus, utilizing the multiscale modelling [77], the membrane protein of interest may first be simulated in a mixed lipid bilayer using CG MD simulations to equilibrate the system and describe how lipids interact on extended timescales, before converting the system to atomistic detail to further refine and characterize the observed lipid-protein interactions. This serial multiscale approach has been successfully used to identify lipid binding sites on membrane proteins [78,79,80,81].

Potential lipid binding sites may also be identified by the computationally cheap, docking calculations. However, these methods do not generally consider the membrane environment in which the interactions occur. Hence the protein-lipid configurations identified by docking may require refinement by subsequent MD simulations.

## 3. Results from Molecular Biophysics on Membrane Proteins—Lipids Interactome

Several biophysical, and functional assays as well as structural methods, have been combined to provide a detailed picture of lipid modulation, e.g., cryo-EM has made a direct way to observe how lipids modulate the different conformations of integral membrane proteins [82,83,84,85].

As mentioned in Section 2.1, the direct, allosteric interactions can be exerted by lipids in non-annular sites that are binding grooves, i.e., interhelical protein interfaces, either intermolecular or intramolecular. It is interesting to show how exactly the modulation of protein function emerges from specific lipid-protein interactions. Lipids can stabilize different conformational states of integral membrane proteins, and structural rearrangements happening within the membrane often imply an active role for the lipids interacting directly, or not, and modulating the energy landscape.

In the case of eukaryotic inward rectifying potassium ion (Kir) channels, initially functional assays revealed that the Kir channels were dependent on the presence of the anionic lipid PIP_2_ for activation. Anionic phosphatidyl lipids in at least three non-annular sites on the potassium channel KcsA from the bacterium Streptomyces lividans are required for channel opening according to piochemical and biophysical assays performed by Lee and collaborators [86]. Docking calculations and AA MD simulations [87] were performed by Sansom and collaborators to identify modulatory anionic PIP_2_ lipid binding sites for potassium channel of streptomyces A (KcsA) using a homology model of inward rectifier potassium channel (Kir) Kir6. Subsequently, both MD simulation studies by Sansom and collaborators [80,88] and crystal structures by MacKinnon and collaborators 44 revealed four specific PIP_2_ binding sites and enabled the structural rationalization of the mechanism of PIP_2_ channel modulation. Rosenhouse-Dantsker and collaborators showed that cholesterol binds in CRAC motifs (see Section 4) in Kir channels [88]. The binding modulated channel function by affecting the hinging motion at the center of the pore-lining TM helix that underlies channel gating either directly or through the interface between the N and C termini of the Kir channel [88].

Docking calculations and AA MD simulations were performed to investigate lipids interactions with Cys-loop receptors. Brannigan and collaborators observed with this combination of methods, cholesterol binding at subunit interfaces of GABA_A_R that possibly promotes pore opening through a wedge mechanism [89].

The nAChR is a pentameric ligand-gated ion channel to both neuronal and muscular processes and is considered the prototype for ligand-gated ion channels. While experimental structures solved by Hibbs revealed the sites for the binding of membrane lipids to pentameric ligand-gated ion channels (pLGICs) the structural basis for the modulation of cation channel nAChR remains still unclear [90]. The underlying cause of nAChR sensitivity to cholesterol was controversial. Docking calculations and AA MD simulations by Klein [91] showed that the nAChR contains embedded cholesterol, i.e., internal sites capable of containing cholesterol, whose occupation stabilizes the protein structure. The MD simulations showed sites at the protein–lipid interface as conventionally predicted from functional data, as well as deeply buried sites that were not usually considered. Both sites most effectively preserved the experimental structure; the structure collapsed in the absence of bound cholesterol. MD simulations showed that bound cholesterol directly supports contacts between the agonist-binding domain and the channel pore that are thought to be essential for activation of the receptor.

CG MD simulations by Sansom were used to characterize in molecular detail the protein-lipid interactions of Kir2.2 channel embedded in a model of the complex plasma membrane. From functional studies and experimental structure it was known that PIP_2_ headgroup interacting with both the transmembrane (TM) domain (TMD) and the cytoplasmic domain, bringing them closer together and favoring a channel open conformation (PDB ID 3SPI [44]). A secondary anionic lipid site has been identified [92], which augments the activation by PIP_2_ while cholesterol inhibits the channel. Kir2.2 has been simulated with multiple, functionally important lipid species. From the simulations it was showed that PIP_2_ interacts most tightly at the crystallographic interaction sites, outcompeting other lipid species at this site.

The role of lipids in the oligomerization of membrane protein complexes is also well known [93,94] as high-resolution structures of oligomeric integrated membrane proteins have occasionally captured lipids in the interface of the monomeric unit of oligomers [68,95,96,97,98,99,100,101,102,103,104,105,106,107,108,109,110,111].

## 4. GPCR—Lipids Interactome

Lipid environment in plasma membrane can modulate the function of GPCRs by indirect or direct interactions with the receptor [28,112].

Plasma membranes concentrate 80–90% of the total cell cholesterol content. Cholesterol is highly abundant in the cell membrane (34% of the total lipid content in mammalian plasma membranes) and some GPCRs are enriched in cholesterol-rich domains. Thus, cholesterol is known to regulate several aspects of GPCRs structure and function [30,113] including ligand binding activity [114], activation [115], signaling [116,117], oligomerization [117,118], and may be a prime regulator of GPCRs, keeping their basal activity low by stabilizing their inactive or intermediate active conformation [119].

The effects of cholesterol to GPCRs may be indirect [112,120] and can originate from cholesterol-mediated changes in membrane properties, e.g., membrane fluidity or can be due to specific, direct interactions. The direct interactions of the non-annular lipids, including cholesterol, with the receptor can be allosteric [29,30,45,73,121] (the lipid binds to a site on the receptor that is separate to where the signal molecule binds) on protein structure which can be weak and very dynamic.

Indeed, the first structural evidence for site-specific cholesterol binding in GPCRs was provided in 2007 by the X-ray structure of the complex between the inactive β_2_AR with carazolol (PDB ID 2RH1 [122]) in which cholesterol binds in distinct cavities of GPCR formed by TMs in different locations. The X-ray structure was solved by Stevens and Kobilka in 2007.

It has been suggested that general allosteric binding sites existed such as the cholesterol Consensus Motif (CCM) in GPCRs, [112,123] initially observed by Papadopoulos and collaborators in benzodiazepine receptor [124], but also to other membrane proteins e.g., by Barrantes and collaborators in nAChR [125], comprising by four amino acid side chains distant in primary sequence [4.39-4.43(R,K)]–[4.46(I,V,L)]–[4.50(W,Y)]–[2.41(F,Y)]. Thus, CCM motifs have been observed by Stevens and collaborators in the X-ray structure of inactive β_2_AR (PDB ID 3D4S [126]) and confirmed by AA or CG MD simulations in this and other GPCRs. Specific binding sites that have high cholesterol affinity for the receptor may constitute a favorable environment for lower-affinity and annular cholesterol molecules. Multiple sequence alignments suggested that this CCM extends far beyond β_2_AR to include as many as 44% of human GPCRs. However, even if the CCM motif is conserved in 44% of human class A GPCRs, it does not always correlate with cholesterol binding sites observed in high resolution crystal structures or MD simulations. Cholesterol Recognition Amino Acid Consensus (CRAC) motifs were also identified as contiguous residue sequences localized to single ΤΜ helices. CRAC motifs have the sequence -L/V–(X)_1-5_–Y/F–(X)_1-5_–R/K- determined based on the sequence or in a crystal structure and suggested to stabilize GPCRs in the bilayer. A CRAC motif has been calculated in TM7, co-localized with the highly conserved NPxxY motif, found conserved in 38% class A GPCRs [72]. The presence of CRAC motifs in a transmembrane region suggests the “possibility” of cholesterol binding with the receptor.

In other studies, it was observed that a lipid can perturb the ligand binding pocket directly. From an extensive set of 14 μs-AA MD simulations (0.25 ms total duration) [55] of active β_2_AR in 1-hexadecanoyl-2-(9Z-octadecenoyl)-sn-glycero-3-phospho-(1′-rac)-glycerol or 1,2-palmitoyl-oleoyl-sn-glycero-3-phosphoglycerol (POPG) bilayer was performed by Garcia and collaborators. In this work [55] it was possible to observe an anionic PG lipid entering the core of the activated β_2_AR laterally via an opening between the cytoplasmic portions of helices TM6 and TM7. Once bound the PG molecule formed electrostatic interactions with the protein which inhibited the formation of the ionic lock, a key interaction thought to stabilize the inactive state of the receptor. Entry of the PG lipid thus led to an increase in stability of the active state of the transmembrane domain, providing a testable mechanism which may explain the experimental observation that anionic lipids can enhance the activity of certain GPCRs [127] including the β_2_AR [48].

However, in another case [128] Voth and collaborators performed 3 μs-AA MD simulations in A_2A_R in the apo and its ZM241385 bound conformation (PDB ID 3EML [129]) embedded in POPC bilayer and showed an opening of a cleft between TM1 and TM2 allowing entry of a lipid headgroup into the binding pocket, perhaps contributing to inactivity of the receptor.

Similarly, Selent and Martín in 1μs-AA MD simulations [48] of the inactive A_2A_ adenosine receptor (A_2A_R) bound with antagonist ZM241385 (PDB ID 3EML [131) embedded in POPC bilayer observed a spontaneous entry of a cholesterol molecule from the membrane-phase into the orthosteric ligand binding pocket.

## 5. Modulation of Adenosine A_2A_ Receptor by Membrane PIP_2_ Lipids and Cholesterol

### 5.1. The Adenosine A_2A_ Receptor

A_2A_R is a G_s_ coupled receptor activated by the naturally occurring adenosine G_s_ protein activates enzyme adenylyl cyclase (AC) that produces cyclic adenosine monophosphate (cAMP). The signaling pathways used by the A_2A_R depend on the type of cell and tissue where the receptor is localized, the specific G effector protein to which it is coupled, and the signaling machinery that the cell possesses [130].

Human A_2A_R is one of the best structurally characterized GPCRs, with more than 54 structures resolved experimentally (see Supporting Information in ref. [131]). The comparison between the experimental structures of the inactive state of A_2A_R bound to antagonists or A_2A_R bound to agonists and the active state of A_2A_R bound to agonist and a G protein as well as the investigation of such structures with MD simulations [132] have revealed characteristics of the conformational changes occurring during receptor activation by bound agonists. Human A_2A_R has been at the forefront of drugs acting against Ars [130].

**Figure 1:**
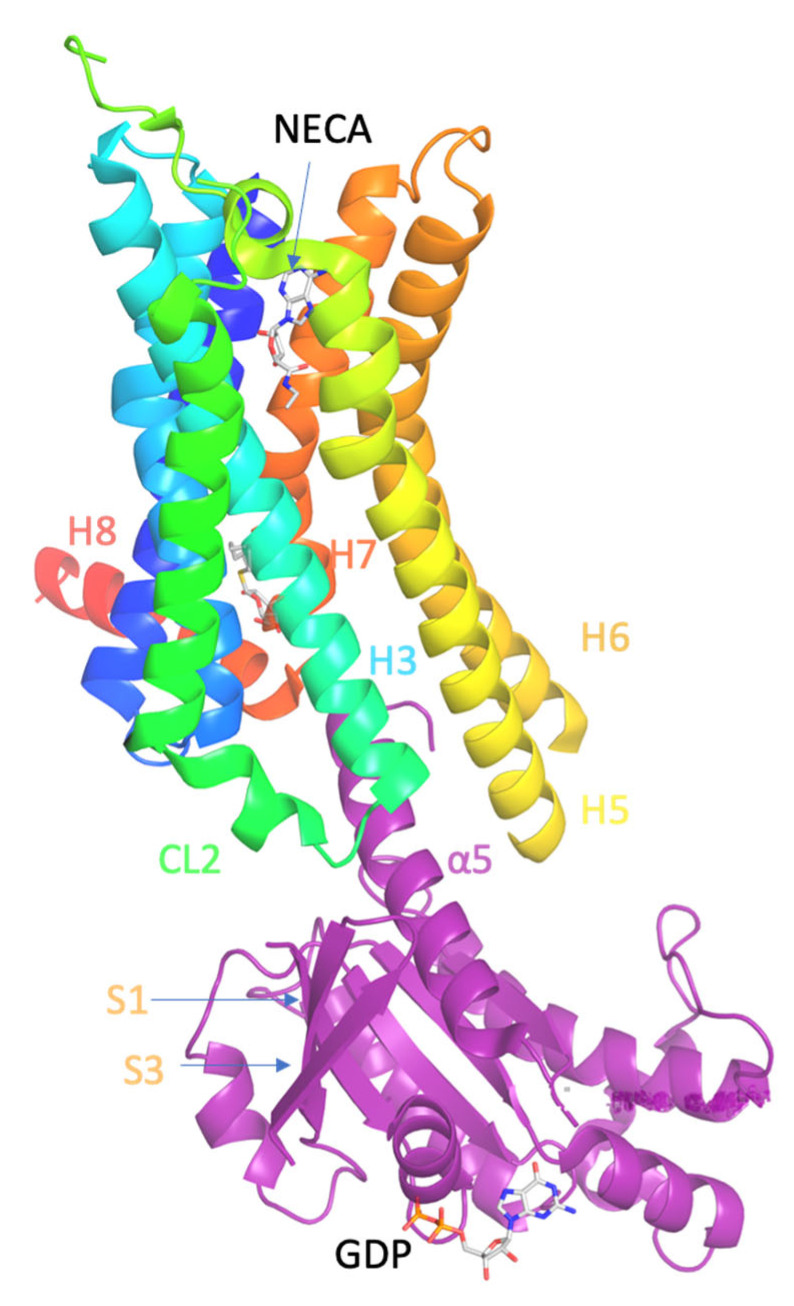
Ligand binding and overall structure of the A_2A_R–mini-G_s_ complex (PDB ID 5G53 [139]). The structure of A_2A_R is depicted as a cartoon in rainbow coloration (N-terminus in blue, C-terminus in red) with mini-G_s_ in purple. The agonist NECA bound to A_2A_R and GDP bound to mini-G_s_ are depicted as stick models (carbon, white; nitrogen, blue; oxygen, red; phosphorous, orange). Relevant secondary structural features are labelled.

Thus, A_2A_R has been extensively studied over the last few decades and its complexes with agonists [133,134], like adenosine (PDB IDs 2YDO [134]) or 5′-N-ethylcarboxamidoadenosine (NECA; PDB IDs 2YDV [134]) and several antagonists [129,135,136,137,138], e.g., with ZM241385 (PDB ID 4EIY [136]) have been solved with X-ray crystallography. The X-ray structures of inactive state of A_2A_R in complex with antagonist ZM241385 were solved by Stevens (PDB ID 3EML [129]) or by Stevens and Cherezov (PDB 4EIY) and in complex with agonists (PDB IDs 2YDO [134]) by Tate and collaborators. The X-ray structure of A_2A_R in a complex with mini-G_s_ protein and the agonist NECA has been solved by Tate and collaborators at 3.4 Å resolution and is the only structure available for active A_2A_R (PDB ID 5G53 [139]), see Figure 1. This structure was reported 5 years after the agonist-bound β_2_AR-Nb80 (PDB ID 3P0G [140]) was solved by Kobilka and collaborators.

The crystal structure of A_2A_R–mini-G_s_ complex bound to agonist NECA (PDB ID 5G53 [139]) showed that the intracellular side of the GPCRs interacts with the N- and C-terminal α-helices of G protein, i.e., the Gα protein (Figure 1). Compared with the inactive state of A_2A_R formed in complex with an antagonist, e.g., ZM241385 (PDB ID 3EML [129] or 4EIY [136]), the active A_2A_R is found to undergo significant conformational changes upon agonist activation and G protein binding.

The largest conformational change, which was initially proposed for the β_2_AR by Gether [141], in the activation procedure of A_2A_R [139] consists by the breakage of the conserved ionic lock in the central salt-bridge between R102 [48,142] in TM3 and E228 [4,28] in TM6. This leads to the ~14 Å movement of the C_α_ of W224 [4,24] or E228 [4,28] at the cytoplasmic end of TM6 to accommodate G protein binding, as shown by the comparison between active and inactive forms of A_2A_R. In comparison, in A_2A_R with only the adenosine agonist bound (without the G_i_ bound), resulting in the so-called the intermediate-active conformation (PDB ID 2YDO [134]) the outward movement at the cytoplasmic end of TM6 is ~11 Å. In contrast to the considerable re-arrangements of the cytoplasmic half of the receptor, no significant changes were observed in the extracellular half of the receptor with exception of a comparatively subtle change in the orthosteric ligand-binding pocket.

Single-molecule Förster Resonance Energy Transfer (smFRET) experiments were performed on functionally active human A_2A_R molecules embedded in freely diffusing lipid nanodiscs to study their intramolecular conformational dynamics [143]. A dynamic model of A_2A_R activation was suggested that involves a slow (>2 ms) exchange between the active-like and inactive-like conformations in both apo and antagonist-bound A_2A_R, explaining the receptor’s constitutive activity.

Ιn the receptor’s active state, agonist binds to the orthosteric site of A_2A_R which in turn binds to the G protein (e.g., G_s_) causing the exchange of guanosine diphosphate (GDP) for the guanosine triphosphate (GTP) bound to the Gα subunit and the dissociation of the Gβγ heterodimer. The activation of G_s_ (or other G) protein that results in increased concentration of cAMP is the major general pathway of A_2A_R activation. The activated Gα,s stimulates adenyl cyclase (AC) type VI, which increases levels of cAMP in cells activating protein kinase A (PKA); the latter phosphorylates and stimulates cAMP responsive element binding protein 1 (CREB1). The activation of A_2A_R triggers activation of several other kinases, e.g., the mitogen-activated protein kinases (MAPK) and extracellular signal-regulated kinases (ERK) reported by Fredholm and collaborators [144]. Phosphorylation of some of the kinases lead to specific cellular responses. Additionally, Gs (or other G) protein may stimulate the formation of phospholipase C (PLC). PLC is an enzyme which hydrolyzes PIP_2_ into inositol 1,4,5-trisphosphate (IP3) and diacyl glycerol (DAG). In turn, IP3 and DAG cause Ca^2+^ release from the ER and PKC activation, respectively. Gαq and some Gβγ complexes can also activate PLC.

### 5.2. A_2A_R—PIP_2_ Interaction

#### 5.2.1. Experimental Findings for A_2A_R—PIP_2_ Interaction

Using high resolution native mass spectroscopy and CG MD simulations by Robbinson C, Sansom and collaborators the endogenous lipid–receptor interactions A_2A_R were investigated [145].

**Figure 2:**
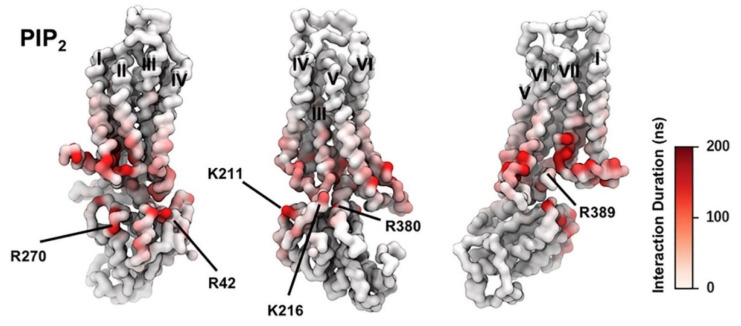
PIP_2_ Interactions with A_2A_R + mini-G_s_ Complex; the duration of PIP_2_ interaction with A_2A_R in active + mini-G_s_ state is mapped onto the receptor structure shown in three different orientations. Major interacting residues on mini-G_s_ are labeled (adapted with permission from Ref. [146] 2019 Elsevier).

PIP_2_ lipids were observed bound directly to the trimeric G_αsβγ_ protein complex of the adenosine A_2A_R in the gas phase using mass spectroscopy. The presence of PIP_2_ at the interface between the receptor and mini-G_s_ in the PMF calculation implies that PIP_2_ molecules form bridging interactions with positive charged residues in the Gα surface (e.g., R42, R270, R380, R389, K211, K216) and in the TM6/TM7 edge (K233^6.35^, R291^7.56^, R293^8.48^, R296^8.51^]) to stabilize the complex (Figure 2). It was observed the preferential binding of the anionic phospholipids PIP_2_ in A_2A_R over related endogenous phospholipids, e.g., phosphatidyl serine (PS), and confirmed that the intracellular surface of the receptors contains hotspots for PIP_2_ binding. The stabilizing interactions of anionic PIP_2_ lipids between TM6 and TM7 and G_α_ (α5) may favor the outward movement of the cytoplasmic half of TM6 that is characteristic of GPCR activation.

In another study by Eddy and collaborators, using ^19^F NMR spectroscopy [147] of the A_2A_R receptor in n-dodecyl-β-D-maltopyranoside (DDM)/cholesteryl hemisuccinate (CHS) mixed micelles and nanodiscs consisting by POPC mixed with one type of anionic lipid, including POPS, 1,2-palmitoyl-oleoyl-sn-glycero-3-phosphate (POPA), POPG, or PIP_2_, it was shown that anionic phospholipids prime the receptor to form complexes with mini-G_αS_ protein through a conformational selection process without demonstrating a particular influence of the unique polar phospholipid heads. A variation in dissociation constants (K_D_) values of the antagonist ZM241385 and agonist NECA among different lipid compositions by a factor of ~2 and ~3, respectively, also showed no obvious correlation between lipid headgroup and determined K_D_ value [147]. Without anionic lipids, signaling complex formation proceeds through a less favorable induced fit mechanism. In computational models, the anionic lipid mimic interactions between a G protein and positively charged residues in A_2A_R stabilizing a pre-activated receptor conformation. Replacing these residues in the intracellular part of A_2A_R strikingly alters the receptor response to anionic lipids in experiments [147].

#### 5.2.2. MD Simulations Findings for A_2A_R—PIP_2_ Interaction

CG MD simulations using an in vivo-mimetic membrane were performed by Sansom and collaborators [145,146] to describe the stabilizing electrostatic interactions of anionic phospholipids with the positively charged amino acids of A_2A_R-Gs interface. Sansom and collaborators performed 10 repeats of 8 μs CG MD simulations [146] of A_2A_R.

**Figure 3:**
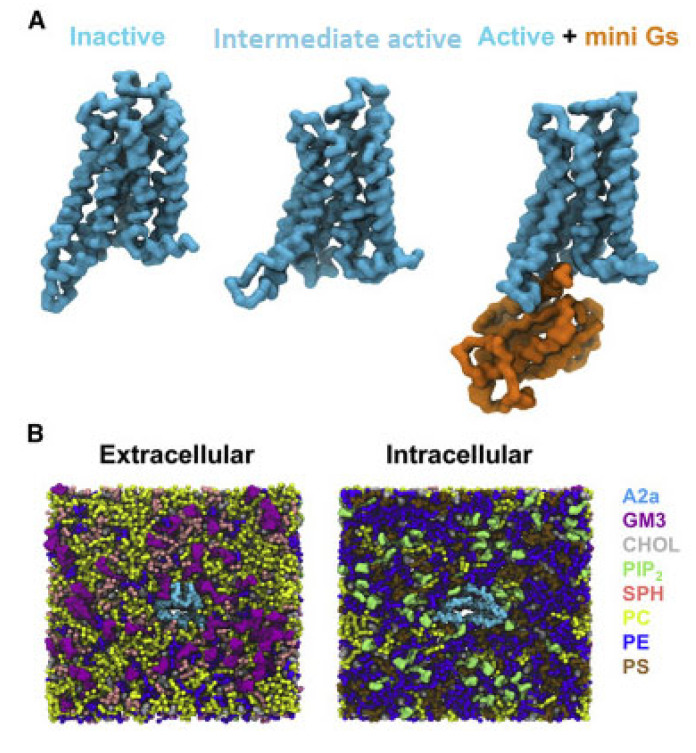
CG model in in vivo-mimetic membrane. (**A**) Three different conformational states (inactive, PDB: 3EML; active, PDB: 5G53, subunit A; and active + mini-Gs, PDB: 5G53, subunits A and C) of the A2aR used in the simulations. (**B**) An overview of the simulation system from the extracellular and intracellular sides. The receptor is colored cyan and different lipid species are colored as specified (adapted with permission from Ref. [146] 2019 Elsevier).

For the simulations it was used the X-ray structures of inactive A_2A_R bound with antagonist ZM241385 (PDB ID 3EML [129]), the intermediate state using the X-ray structure of A_2A_R bound with agonist NECA (PDB ID 2YDV [134]), and the active A_2A_R-NECA agonist-mini-G_s_ (PDB ID 5G53 [139]) embedded in plasma mimetic membrane. The membrane bilayer contained POPC (20%): DOPC (20%): POPE (5%): DOPE (5%): SPH (15%) GM3 (10%): cholesterol (25%) within the upper leaflet, and POPC (5%): DOPC (5%): POPE (20%): DOPE (20%): POPS (8%): DOPS (7%): PIP_2_ (10%): cholesterol (25%) within the lower leaflet (Figure 3).

A key finding from the mass spectroscopy experiments [145] and CG MD simulations [145,146] was that the polyanionic lipid PIP_2_ enhanced the interaction between the A_2A_R and a mini-G_s_ protein.

Additionally, as reported in ref. [145] anionic phospholipids PIP_2_ bind more tightly in A_2A_R-Gs interface over related endogenous phospholipids, e.g., phosphatidyl serine (PS). Indeed, comparison of the potential of mean force with umbrella-sampling PMF (US) CG MD calculations for PIP_2_-bound versus PS-bound receptor in a lipid bilayer indicates that the interaction of mini-Gs with A_2A_R is stabilized significantly (~50 ± 10 kJ/mol) in the presence of PIP_2_ compared with PS [145].

**Figure 4:**
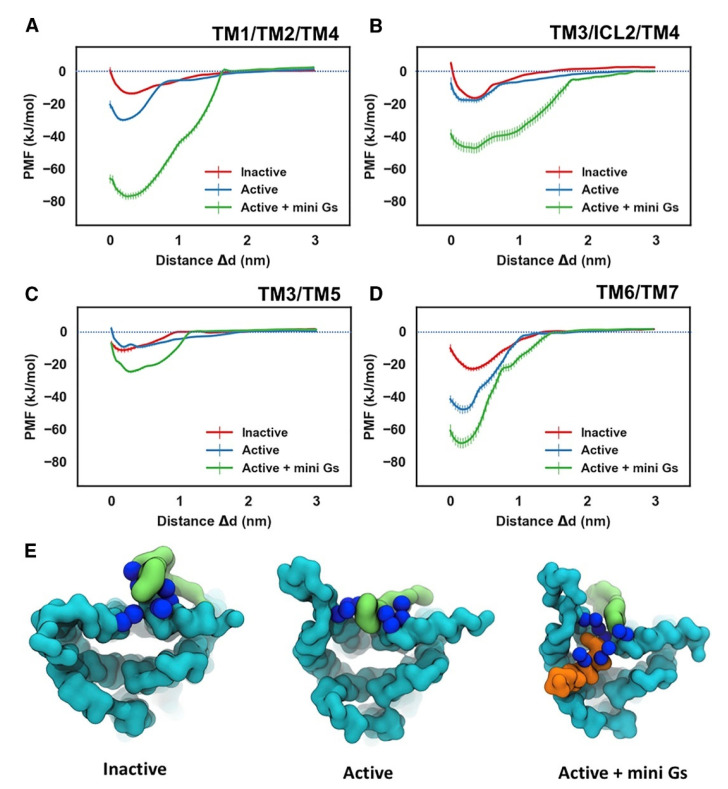
Energetics of PIP_2_ Interaction with A_2A_R. PMFs for PIP_2_ binding to the sites defined by TM1/TM2/TM4 (**A**), TM3/ICL2/TM4 (**B**), TM3/TM5 (**C**), and TM6/TM7 (**D**). The PMFs from the simulations of PIP_2_ bound to the inactive state, active state, and active + mini-G_s_ state of the receptor are colored in red, blue, and green, respectively. PIP_2_ bound to the TM6/TM7 site in the three conformational states is shown in (**E**) viewed from the intracellular side of the receptor. The receptor, the bound PIP_2_ molecule, and the G_α_ α5 helix are colored in cyan, green, and orange, respectively. The basic residues that form the binding site of TM6/TM7 (K233^6.35^, R291^7.56^, R293^8.48^, R296^8.51^) are shown as blue spheres (adapted with permission from Ref. [146] 2019 Elsevier).

PIP_2_ molecules bound to cationic intracellular edge on the A_2A_R and formed an extended anionic surface at the cytoplasmic face of the receptor which facilitates the recruitment of G protein via formation of bridging interactions with basic residues on G_a_ (Figure 2 and Figure 4E). Additionally, the CG MD simulations showed that PIP_2_ binds to TM3/ICL2/TM4 and TM3/TM5, TM1/TM2/TM4 and TM6/TM7 [146]. The PMF (US) CG MD simulations [146] showed that PIP_2_ binds to TM3/ICL2/TM4 and TM3/TM5 with equal strength between the inactive and active states of the receptor (Figure 4B,C). However, for the TM1/TM2/TM4 (Figure 4A) and TM6/TM7 (Figure 4D) sites, there was significantly stronger binding of PIP_2_ to the receptor in the intermediate active state (agonist complex) and the active (agonist with mini-G_s_ complex) state than to that in the inactive state of the A_2A_R, especially for the TM6/TM7 site at which an increase of 23 kJ/mol in binding strength was observed (Figure 4D). The outward movement of TM6 that is required for GPCR activation and G protein association opens the intracellular side of the receptor allowing PIP_2_ to bind more deeply and tightly in this site [146].

### 5.3. A_2A_R—Cholesterol Interaction

#### 5.3.1. Experimental Findings for A_2A_R—Cholesterol Interaction

Cholesterol has been shown to be necessary for the activation of A_2A_R. In a study by Robinson A [148] with mammalian cells expressing adenosine A_2A_R agonist triggered downstream signaling (shown through production of cAMP) which was found to be reduced following membrane cholesterol depletion from the cell membrane, with methyl-beta-cyclodextrin (MβCD) [148]. The in vitro activity of purified receptors was affected by alterations to cholesterol concentrations, as seen by ablation of radioligand binding for purified A_2A_R without cholesteryl hemisuccinate (CHS) in a work by Martín, Selent and collaborators [56]. These findings suggested that A_2A_R signaling is dependent on cholesterol and contradicted previous suggestions that cholesterol negatively modulates A_2A_R [148]. In contrast, ligand (agonist or antagonist) binding affinity was not dependent on cholesterol depletion [148] although experiments employing radioligand-binding assays on A_2A_R showed that cholesterol significantly decreases the binding of the antagonist to the receptor [56].

**Figure 5:**
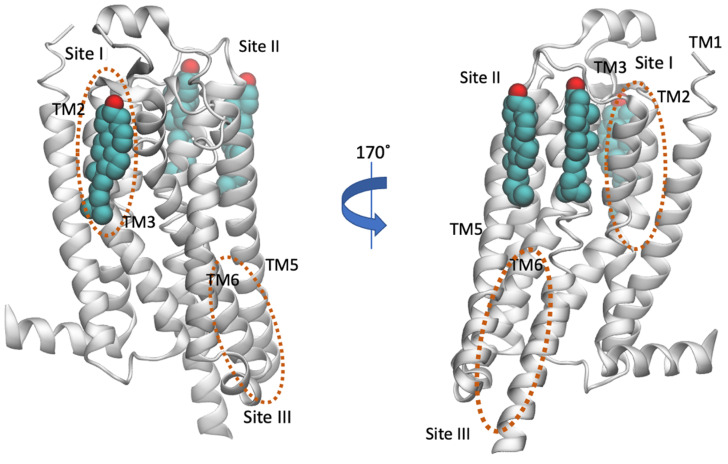
Location of cholesterol molecules (shown as van der Waals spheres) that were resolved in the crystal structure in inactive A_2A_R (PDB ID 4EIY [136]) is shown. The experimental sites are positioned to the extracellular part of the receptor, in the regions TM2-ECL1-TM3 (Site I) and TM5/ECL3/TM6, TM6-TM7 (Site II) (for representative PDB IDs see e.g., references [129,136,149,150,151,152,153,154,155,156,157]). The cholesterol binding regions calculated by MD simulations were observed in the extracellular TM6/TM7 (Site II with AA MD simulations [158] or CG MD simulations [146]) and in the intracellular TM4 (Site III, CG MD simulations [74]) and are encircled with an orange line. Site III has not been observed experimentally.

As reviewed by Moreau [131], A_2A_R has a sufficiently large number of structures (54) where the presence and position of cholesterol molecules can be compared. These structures suggested specific dynamics of cholesterol molecules correlated with the type of the ligand, with antagonists increasing the number of bound cholesterols (e.g., in PDB ID 4EIY [136]) without specificity for the ligand in the orthosteric binding site. Thus, in the presence of agonists without G_i_ protein, 9 structures were obtained (e.g., the intermediate active A_2A_R bound to adenosine with PDB ID 2YDO [134]) and showed no cholesterols [133]. In contrast, several X-ray structures of A_2A_R-antagonist complexes have been solved (for representative PDB IDs e.g., see refs [129,136,149,150,151,152,153,154,155,156,157]) with three or four cholesterol molecules bound to the extracellular part of the receptor, in the regions TM2-ECL1-TM3 (site I), TM5/ECL3/TM6, TM6-TM7 (site III), shown in Figure 5. Thus, the direct binding sites of lipids to GPCRs can be weak and very dynamic or can correspond to tighter binding as has been observed for cholesterol in the inactive A_2A_R conformations bound with antagonist, e.g., with ZM241385 (PDB ID 4EIY [136]).

As previously mentioned, the presence of CCM is defined for 44% of human class A GPCRs that implied specific cholesterol binding as important to the structure and stability of class A GPCRs, and that such sites may provide targets for therapeutic discovery.

However, in some GPCRs may not show cholesterol in the presence of CCM due to receptor modifications necessary for stabilizing the receptor prior to crystallization. An example was given with mutation K122_4.43_A used for crystallization studies of A_2A_R [159] since K122^4.43^ as part of CCM can interact with cholesterol and its alteration to alanine can change structured cholesterol binding site. K122^4.43^ is a component of a thermostabilized, antagonist-favored variant of A_2A_R [159]. Indeed, in a subsequent functional assay study [160] it was shown that K122_4.43_A mutation of the wild type (WT) A_2A_R in the intracellular leaflet produced reduction by 2-fold in both agonist and antagonist affinity. Membrane cholesterol depletion by MβCD experiments with K122_4.43_A A_2A_R [160] demonstrated that cAMP concentrations decreased, suggesting cholesterol still affects receptor activity when K122^4.43^ is changed to alanine possibly due to other binding sites, described in Figure 5.

These results suggested that cholesterol modulates A_2A_R cAMP activation through specific interactions at the CCM in a state-dependent manner. However, the CCM was not observed in the X-ray structures.

Additionally, K122_4.43_A A_2A_R binding to G_as_, measured as a decrease of agonist-induced cAMP formation, was significantly decreased compared to A_2A_R WT. Furthermore, as K122_4.43_A showed a modest decrease in agonist and antagonist affinity compared to A_2A_R WT, the decrease in agonist-induced cAMP suggested cholesterol association to K122^4.43^ and an overall significant effect on functional A_2A_R W129_4.50_A states. When K122^4.43^ was mutated to alanine this cholesterol interaction might prevent signaling.

These results suggested that cholesterol modulates A_2A_R cAMP activation through specific interactions at the CCM in a state-dependent manner.

#### 5.3.2. MD Simulations Findings for A_2A_R—Cholesterol Interaction

The interactions between A_2A_R and cholesterol has been studied by various groups including Voth and collaborators [128], Lyman and collaborators [74,148,158,161,162], Selent, Martin and collaborators [56], Lovera, Sands and collaborators [163], Lee, Essex and collaborators [71] Sansom and collaborators [146], Kolocouris and collaborators [164], using both AA MD simulations [56,74,128,148,158,161,163] (or AA models from back mapping the CG models [164]) and/or CG MD simulations [73,76,143,148,161,162] and the inactive [56,71,148,158,161,163,164], intermediate active [71,74,148,161,162] or active A_2A_R [146,164].

**Figure 6:**
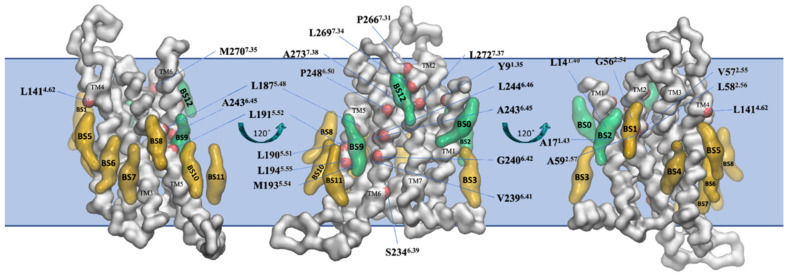
Binding sites of cholesterol in the thirteen distinct binding sites (BS0-BS12) for the inactive state of A_2A_R in plasma mimetic membrane. Binding sites were identified after the analysis of the last 8 μs of the 10 μs-CG MD simulations. The receptor is shown in white surface and representative cholesterol binding sites are shown in green surface (residence time in these binding sites is more than 1 μs) or yellow surface (residence time in these binding sites is less than 1 μs). Residues that belong to the identified binding sites with more than 1 μs cholesterol residence time are shown with a red surface (reproduced from Ref. [164]).

The final snapshot from the 10 μs-CG MD of the inactive A_2A_R embedded in phospholipid bilayers from Kolocouris and collaborators [164] is shown in Figure 6.

**Figure 7:**
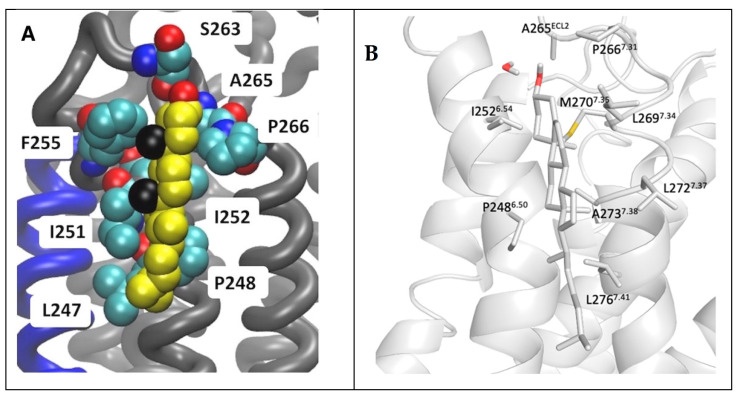
Location of cholesterol molecule (shown as van der Waals spheres) in extracellular leaflet between TM5/TM6 (Site II in Figure 5) on the inactive A_2A_R bound with antagonist ZM241385 (PDB ID 4EIY [136]). (**A**) Snapshot from 6 μs AA MD simulations of inactive A_2A_R embedded in DPPC/DOPC/cholesterol 55:15:30. Cholesterol is shown as van der Waals spheres and receptor in liquorice (adapted from Ref. [74]). (**B**) Last snapshot from 10 μs CG MD simulations of inactive A_2A_R embedded in plasma membrane after back mapping to an atomistic model. The receptor is shown in white cartoon; cholesterol and interacting residue side chains are shown in sticks (adapted from Ref. [164]).

A binding site of cholesterol in the extracellular membrane leaflet obtained from the AA MD of the inactive A_2A_R embedded in phospholipid bilayers from Lyman and collaborators [74] is shown in Figure 7A. The AA model of a cholesterol persistent binding site (BS12) obtained after back mapping the last snapshot from CG MD simulations is shown in Figure 7B.

As is shown in Figure 7 it was predicted the same cholesterol binding along TM6 in the extracellular membrane leaflet (see site II, Figure 5) as observed in the X-ray structures (for representative PDB IDs of inactive A_2A_R structures see e.g., Refs. [129,136,149,150,151,152,153,154,155,156,157]). Site III shown in Figure 5 which has not observed experimentally was predicted in both works also as a prevalent cholesterol binding position in inactive A_2A_R.

**Figure 8:**
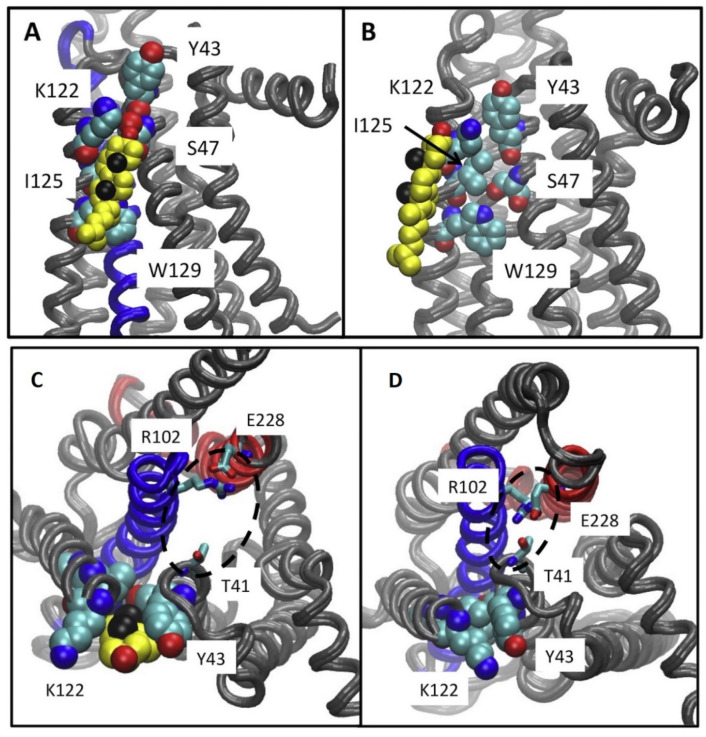
(**A**–**D**) Results from 6μs-AA MD simulations. (**A**) Location of cholesterol molecule (shown as van der Waals spheres) in intracellular CCM between TM2-TM4, with contact residues Y43^2.41^, S47^2.45^, K122^4.43^, I125^4.46^, W129^4.50^ in the intermediate active A_2A_R structure. (**B**) Location of cholesterol molecule (shown as van der Waals spheres) along intracellular TM4, interacting only with K122^4.43^, I125^4.46^ in the intermediate active A_2A_R structure. This bound cholesterol does not lie in CCM to break the ionic lock E228^6.30^-R102^3.50^. Neither position have been found in experimental structures of the intermediate active or inactive A_2A_R. (**C**,**D**) Ionic lock interactions with and without cholesterol at the CCM are shown. (**C**) It is shown a typical state of the ionic lock when cholesterol is bound at the CCM, in which T41 (1/2 turn of helix removed from the Y43 of the CCM) does not participate in the ionic lock interaction network. (**D**) A tight interaction between the residues of the ionic lock is shown, including T41 from helix 2 and R102 and E228 from the conserved ionic lock motif in the experimental structures of inactive A_2A_R (adapted from Ref. [148]).

Lyman and collaborators using AA MD simulations of the intermediate active A_2A_R suggested that cholesterol lies in an intracellular CCM in a cleft between TM2-TM4 in the intracellular membrane leaflet. Cholesterol interacts with residues Y43^2.41^, S47^2.45^, K122^4.43^, I125^4.46^, and W129^4.50^ (Figure 8) of the intracellular membrane leaflet [148]. The tyrosine and lysine residues of the CCM (Figure 8) were positioned in the MD simulations to form hydrogen bonds with the hydroxyl group in cholesterol, while the isoleucine residue could form hydrophobic contacts with cholesterol. The tryptophan residue was predicted to form a ring stacking interaction with the ring in cholesterol.

It has been suggested that this intracellular cholesterol CCM in TM2-TM4 which was not present in experimental structures of the intermediate active inactive A_2A_R complexed with ZM241385 (e.g., PDB ID 2YDV [134]) may facilitate the ionic lock E228^6.30^-R102^3.50^ break towards active state formation (Figure 7C,D). As mentioned previously another study Robinson A. and collaborators performed mutagenesis and functional assays [160] with, K122_4.43_A, W129_4.50_A A_2A_Rs. They suggested [160] that cholesterol bind to a CCΜ in the intermediate active state A_2A_R forming stabilizing interactions with W129^4.50^ and K122^4.43^ supporting findings in Refs. [148,162].

This CCM has been also identified experimentally in the inactive state of β_2_AR bound to partial inverse agonist timolol (PDB ID 3D4S [126]). A cholesterol bound to intracellular TM4 had not been observed also in experimental structures of inactive A_2A_R (e.g., PDB ID 4EIY [136]).

## 6. Modulation of Other GPCRs by Membrane PIP_2_ Lipids and Cholesterol

Phospholipids and cholesterol are known to bind to GPCRs to modulate their activity [30] through direct interactions that can affect functional activity of a receptor with two mechanisms.

(a) A lipid can bind in an allosteric position. As mentioned previously, the first structural evidence for directly bound cholesterol binding in allosteric positions in GPCRs was provided in 2007 by X-ray structures of the complex between the inactive dimeric β_2_AR with carazolol (PDB ID 2RH1 [122]) and the complex between the monomeric inactive β_2_AR with timolol (PDB ID 3D4S [126]). Then, cholesterol bound was observed in the X-ray structures of the inactive A_2A_R with antagonist ZM241385 and later in structures of complexes with other antagonists [129,136,149,150,151,152,153,154,155,156,157] (e.g., PDB ID 4EIY [136], 5IU4 [157]) in which cholesterol binds in distinct cavities of GPCR formed by TMs in different locations.

A putative cholesterol binding CRAC sequence was reported for transmembrane helix 7 of human CB_1_ cannabinoid receptor (CB_1_R) receptor (CB_1_R) by Maccarone and collaborators [165]. This sequence was proposed to be involved in directing the interaction of CB_1_R with cholesterol-rich microdomains of cell membranes. Moreover, the presence of a cholesterol molecule was reported in the X-ray structure of CB_1_R-AM6538 complex (PDB ID 5XRA [166]) solved by Liu, Stevens Makriyannis, Bohn and collaborators and in cryo-EM structure of CB_1_R-Gi-MDMB-Fubinaca (FUB) agonist (PDB ID 6N4B [167]) solved by Skiniotis, Kobilka and collaborators. At the same time, there was no evidence of a specific retention of cholesterol in the X-ray structure of CB_2_ cannabinoid receptor (CB_2_R)–antagonist AM10257 complex (PDB ID 5ZTY [168]) solved by Liu and collaborators or the CB_2_R-Gi-agonist WIN 55,212-2 signaling complex (PDB ID 6PT0 [169]) solved by Xie, Xu, Zhang and collaborators.

(b) Perturbation of the ligand binding pocket directly, as has been observed for cholesterol binding in the cryo-EM structure of the complex of serotonin (5-hydroxytryptamine, 5-HT) with its type 1A receptor (5-HT_1A_R; PDB ID 7E2Z [47]) solved by Xu, Zhang, Jiang and collaborators. In the structure of 5-HT_1A_ in complex with the agonist aripiprazol (PDB ID 7E2Z [47]), one cholesterol molecule that was inserted into a cleft between TM1 and TM7 is found to be involved in the shaping of the ligand pocket by stabilizing the positions of TM1 and TM7. This is consistent with the central role of cholesterol in the functional regulation of 5-HT_1A_. Additionally, studies have suggested lipid molecules may exit the bulk lipid phase of the membrane, and laterally reach annular and non-annular lipids position or even enter the core of GPCR as was observed in the MD simulations for the inactive state of A_2A_R [56,128].

(c) The non-annular lipids can also act on GPCR-G_i_ interface (which is also an allosteric modulation). Examples are provided by the PIP_2_—A_2A_R interaction described in Section 5.2. Additionally, it has been reported by Xu, Zhang, Jiang and collaborators on the tight interactions of phosphatidylinositol 4-phosphate (PtdIns4P) with positively charged amino acids of 5-HT_1A_R and G proteins in the 5-HT_1A_R-G interface for 5-HT_1A_R [47]. PtdIns4P and cholesterol have been observed on GPCR-G_i_ interface, between TM6 and G α5 helix, in experimental structures of 5-hydroxytryptamine (serotonin) in complex with serotonin_1A_ receptor (5-HT_1A_R; PDB ID 7E2Y [47]) or of agonist aripiprazol with 5-HT_1A_R (PDB ID 7E2Z [47]). PtdIns4P is the precursor of PIP_2_ and has been shown to be a key mediator of GPCR-stimulated production of diacylglycerol, a second messenger [47].

In the structure of 5-HT_1A_R in complex with the agonist aripiprazol (PDB ID 7E2Z [47]), one cholesterol is involved in the shaping of the ligand pocket. In addition, cholesterol is also directly involved in the binding of PtdIns4P to enhance G-protein coupling and signaling activity as shown in the complexes of serotonin with 5-HT_1A_R (PDB ID 7E2Y [47]) or agonist aripiprazol with 5-HT_1A_R (PDB ID 7E2Z [170]).

In another study by Malmstadt and collaborators, 5-HT_1A_R was incorporated in synthetic bilayers of controlled composition together with a fluorescent reporting system that detects GPCR-catalyzed activation of G protein to measure receptor-catalyzed oligonucleotide exchange. The results showed that increased membrane order induced by sterols and sphingomyelin increased receptor-catalyzed oligonucleotide exchange. Increasing membrane elastic curvature stress also increases this exchange [171]. It seems there was a dependence of 5-HT_1A_R on plasma membrane properties suggesting that compositional changes related to aging, diet, or disease could impact cell signaling functions.

In studies of β_2_AR signaling by Kobilka and collaborators, it was shown experimentally that anionic lipids impacted the preference of the receptor to interact with Gα_i_ over Gα_s_ through complementarity of charges between anionic lipids and positive residues in G protein interacting surface [172]. Govaerts and Kobilka also showed that the IC_50_ values for β_2_AR ligands varied among different lipid compositions in nanodiscs by a factor of ~3 for antagonists and ~7 for agonists, though no clear relationship was observed between lipid headgroup type and measured IC_50_ values [48].

Examples of other similar observations in GPCRs are the neurotensin receptor 1 (NTS1) and Gq protein where the affinity of Gαq and Gβ1γ1 to active NTS1R increased with increasing anionic lipid POPG content as was shown by Grisshammer and collaborators [127], the Ghrelin peptide hormone receptor GHSR (growth hormone secretagogue receptor) allosteric modulations by PIP_2_ reported by Banères and collaborators [173].

## 7. Challenges from GPCR-PIP_2_ and Cholesterol Studies

### 7.1. GPCR—PIP_2_ Interaction

The cytoplasmic face of GPCRs undergoes a conserved conformational change to allow coupling of G proteins with the cytoplasmic ends of TM5 and TM6 moving outwards, and TM7 moving inwards slightly [132].

In Refs. [145,146] it was shown that simultaneous binding of the PIP_2_ phospholipid head group to both the G_α_ subunit and TM6 residues stabilize the active G protein-bound states of A_2A_R. This binding of PIP_2_ includes conserved residues, in several class A GPCRs, in parts of the receptors in cytoplasmic phase, e.g., TM5, TM6, H8, ICL1, ICL2. These residues which that are not observed in class B receptors, revealed another role of the cytoplasmic interface of class A GPCRs including the recruitment of PIP_2_ for activation. Examples of such class A GPCRs and examples of their X-ray structures are rhodopsin receptor (with the ground-state chromophore, 11-cis-retinal) solved by Miyan, Stenkamp and collaborators (PDB ID 1F88 [174]), X-ray structure of histamine receptor (H_1_R) in complex with antagonist doxepin (PDB ID 3RZE [175]) solved by Iwata, Stevens, Kobayashi and collaborators, X-ray structure of β_1_AR-antagonist cyanopindolol solved by Schertler, Tate and collaborators (PDB ID 2VT4 [176]), X-ray structure of β_2_AR-carazolol antagonist complex solved by Stevens, Kobilka and collaborators (PDB ID 2RH1 [122]), X-ray structure of CB_1_R–AM6538 antagonist complex (PDB ID 5TGZ [166]) solved by Liu, Stevens Makriyannis, Bohn and collaborators, M4 muscarinic acetylcholine receptor-tiotropium antagonist complex (PDB ID 5DSG [177]) solved by Christopoulos, Kobilka, Sexton and collaborators, A_2A_R-ZM241385 complex (PDB ID 3EML [129]), dopamine D3 receptor (DRD3)–antagonist eticlopride complex solved by Stevens and collaborators (PDB ID 3PBL [178]), sphingosine 1-phosphate receptor- antagonist sphingolipid mimic complex (PDB ID 3V2W [179]) by Stevens, Rosen, Hanson and collaborators.

Different signaling pathways, for example receptor tyrosine kinases or Ca^2+^ signaling, can modulate the local concentration of PIP_2_ in the membrane. The degree of conformational response and activation of GPCRs through differentiation of PIP_2_ concentration in the microdomains of plasma membranes represents another mode of signaling regulation through differentiation of downstream signaling partners in the cell [180].

For therapeutic purposes, synthetic molecules [181] or peptides that bind at the TM5-TM6-TM7 cytoplasmic interface can act as negative allosteric modulators that can inhibit the activation of A_2A_R or other GPCRs by preventing their movement and consequently reducing the affinity of agonists at the orthosteric binding pocket. Otherwise, potent small molecules or peptides [182] can mimic the bridging effects of the PIP_2_ head group and stabilize active states of A_2A_R or other GPCRs.

PIP_2_ is likely to further distinguish binding to β-arrestin as does for G protein subunits between different GPCRs. The declaration of this mechanism can lead to the development of novel biased allosteric agonists that mimic PIP_2_ behavior and bind specifically to the different states of a GPCR that bind to G protein or β-arrestin bound states [183].

### 7.2. GPCR—Cholesterol Interaction

Ιn Ref. [133] it was reported by Moreau the number and position of cholesterol molecules placed between or along TMs and H8 or in interface between dimers (between interfacial TMs, H8 helices). This information was analyzed for the structures of 68 GPCRs in different states (apo, inactive, active and oligomers), ligands (drugs and endogenous agonists or intracellular binders, e.g., G proteins).

Cholesterol has been shown experimentally to be a stabilizer of A_2A_R [148] and it has been observed that the signaling of A_2A_R, coupled to G_αs_ is reduced with cholesterol depletion [148]. This indicated that cholesterol plays an important role in G_αs_ mediated cAMP accumulation, independently of ligand binding stimulation.

There are many A_2A_R experimental structures [131] allowing the comparison of the presence and position of cholesterol molecules. These structures suggested that cholesterol binding was correlated with the type of the ligand, with antagonists binding to A_2A_R increasing the number of bound cholesterols (e.g., in PDB ID 4EIY [136]). In A_2A_R, 41/54 structures with antagonists have at least 2 cholesterols and 31 structures have cholesterol in extracellular membrane leaflet in positions TM2/TM3 (site I in Figure 6), TM6 (close to site II in Figure 6), and often another one cholesterol in TM6 (see site III in Figure 6). The A_2A_R structures with bound agonists and without G proteins (e.g., PDB ID 7ARO [153]) have no or less cholesterols bound compared to the antagonist bound inactive conformations of the receptors (e.g., in PDB ID 4EIY [136]).

An extracellular CCM along Y43^2.41^, S47^2.45^, K122^4.43^, I125^4.46^, W129^4.50^, the last being the most conserved amino acid within the CCM, was suggested by AA MD simulations [162] and functional assays [160] in the intermediate active A_2A_R structure in complex with NECA agonist (PDB ID 2YDV [134]) as that found in the inactive state of β_2_AR (PDB ID 3D4S [126]). Such CCM motifs can be used for designing allosteric antagonists with higher affinity than can displace cholesterol and modulate GPCR function.

Furthermore, there are cases where the mechanism of the allosteric modulation by cholesterol is not known and is useful for allosteric drug design purposes. For example, in the case of CB_2_R the 2-(6-chloro-2-((2,2,3,3-tetramethylcyclopropane-1-carbonyl)imino)benzo[d]thiazol-3(2H)-yl)ethyl acetate ligand (MRI-2646) was shown to act as a partial agonist in membranes devoid of cholesterol and as a neutral antagonist in cholesterol-containing membranes. AA MD simulations based on the cryo-EM structure of the human cannabinoid receptor CB_2_R-Gi-agonist WIN 55,212-2 signaling complex (PDB ID 6PT0 [169]) did not suggest how that cholesterol exerted its allosteric effect on the intracellular regions of the receptor that interact with the G-protein complex thereby altering the recruitment of G protein.

## Data Availability

Not applicable.
